# Dopaminergic, Noradrenergic, Adrenal, and Thyroid Abnormalities in Psychotic and Affective Disorders

**DOI:** 10.3389/fpsyt.2020.533872

**Published:** 2020-09-18

**Authors:** Fabrice Duval, Marie-Claude Mokrani, Alexis Erb, Vlad Danila, Felix Gonzalez Lopera, Ludovic Jeanjean

**Affiliations:** Pôle 8/9-APF2R, Centre Hospitalier, Rouffach, France

**Keywords:** schizophrenia, bipolar disorder, schizoaffective disorder, apomorphine challenge, clonidine challenge, TRH test, dexamethasone suppression test

## Abstract

**Background:**

This study aimed to assess hypothalamic-pituitary dopaminergic (DA), noradrenergic (NA), thyroid (HPT), and adrenal (HPA) activity in schizophrenia, in schizoaffective disorder, and in bipolar disorder.

**Method:**

We investigated a combined approach of hormone responses to (1) apomorphine (APO), a short-acting DA receptor agonist which decreases prolactin secretion (PRL), and stimulates secretion of growth hormone (GH), adrenocorticotropin (ACTH), and cortisol; (2) clonidine (CLO), an alpha 2-adrenoceptor agonist which stimulates GH secretion; (3) 8 AM and 11 PM protirelin (TRH) which stimulates thyrotropin (TSH) secretion; and (4) dexamethasone which suppresses cortisol secretion, in 13 hospitalized healthy male controls and 39 untreated male inpatients: 13 with DSM-IV paranoid schizophrenia, 13 with DSM-IV schizoaffective disorder (bipolar subtype, depressed at the time of the study), and 13 with DSM-IV bipolar disorder (depressed).

**Results:**

Compared to controls, paranoid schizophrenic patients showed (1) lower APO-induced ACTH and cortisol stimulation, and (2) higher post-dexamethasone cortisol values. Compared to controls, schizoaffective and bipolar patients showed (1) lower ΔΔTSH values (i.e., difference between 11 PM and 8 AM TRH-TSH responses), (2) lower APO-induced PRL suppression, (3) lower CLO-induced GH stimulation, and (4) higher post-dexamethasone cortisol values.

**Conclusions:**

Although results must be interpreted with caution because of the small sample, this preliminary study suggests that depressed bipolar and schizoaffective patients share common biological dysregulations, distinct from that of paranoid schizophrenic patients. From a pathophysiological viewpoint, paranoid schizophrenic patients can be characterized by hyposensitivity of the hypothalamic DA receptors (possibly resulting from an increase in presynaptic DA release) associated with increased HPA axis activity, while depressed bipolar and schizoaffective patients can be characterized by hyposensitivity of the pituitary TRH and DA-D_2_ receptors (possibly linked to the activation of the hypothalamic TRH and tuberoinfundibular DA neurons, respectively), together with subsensitive postsynaptic α_2_-adrenoreceptors at the hypothalamic level (possibly secondary to an erratic release of NA) and increased HPA axis activity.

## Introduction

It is now well established that the secretion of the hypothalamic hypophysiotropic hormones is controlled by neurotransmitters posited to play a preeminent role in the pathophysiology of major psychiatric disorders such as schizophrenia (SCH), schizoaffective disorder (SAD), and bipolar disorder (BD) (1, 2). Moreover, significant progress over the last decades has also demonstrated that neuropeptides and neurohormones may be directly involved in numerous mental illnesses [for a review, see (3)]. Thus, the neuroendocrine strategy can characterize the hypothalamic-pituitary dysfunction of affective and psychotic diseases, and assess the functionality of some neurotransmitter systems by using suitable pharmacological stimuli. To evaluate the DA function in psychiatric patients, several studies have used subcutaneous administration of apomorphine (APO), a non-selective short acting dopamine (DA) agonist (4). APO inhibits prolactin (PRL) secretion and stimulates adrenocorticotropic hormone (ACTH), cortisol, and growth hormone (GH) release (4–6). In drug-free SCHs, it has been consistently found blunted hypothalamic-pituitary-adrenal (HPA) axis responses to APO compared to controls (5–8); this blunting may reflect a hyposensitivity of the hypothalamic DA receptors in SCHs. Lower responsiveness of cortisol to APO has also been found in SADs (5); but not in depressed BDs (9). Regarding GH and PRL responses to APO, contradictory results have been reported in SCHs and SADs (4–11). However, some studies found lower APO induced-PRL suppression in depressed BDs compared to healthy controls and unipolar depressed patients (9, 12). Interestingly, it has been reported in patients with major unipolar depressive disorder with HPA axis overactivity and melancholic and psychotic features altered ACTH/cortisol and GH responses to APO (13). These latter findings are in line with the hypothesis that hypercortisolemia by increasing DA release may induce a hyposensitivity of hypothalamic DA receptors (14).

Measurement of GH levels following administration of clonidine (CLO)—a partial α_2_-adrenoceptor agonist—has been widely used in the evaluation of noradrenergic (NA) α_2_-receptor function in psychiatric patients (15). In depressed patients and in SADs, GH response to CLO is often blunted (9, 15, 16) suggesting a hyposensitivity of hypothalamic α_2_-adrenoceptors (15). In SCH, GH response to CLO differs from study to study: increased, decreased, or unchanged responses have been reported [for review, see (3)].

Overactivity of the HPA axis, and increased levels of cortisol, is one of the most replicated biological findings in severe depressed patients (17). However, hyperactivity of the HPA axis is not specific to depression since it has also been found in SCH and SAD (18, 19). Although, the mechanisms underlying this abnormality are not fully understood, the most striking feature is that type II glucocorticoid receptor (GR)-mediated feed back inhibition is impaired—as reflected by a nonsuppression or an early escape of serum cortisol levels in response to the dexamethasone suppression test (DST) (20).

Many euthyroid major depressed inpatients display a chronobiological HPT axis dysregulation (i.e., loss of the nocturnal surge of thyrotropin [TSH], blunted 11 PM TSH response to protirelin [TRH] test, and reduced difference between 11 PM and 8 AM TRH-TSH responses [ΔΔTSH] (21), possibly associated with abnormal morning TRH-TSH response and/or alterations in total and/or free thyroxine (T_4_) and triiodothyronine (T_3_) serum concentrations (22). Chronobiological dysregulation of the HPT axis (as reflected by reduced ΔΔTSH values) has rarely been found in SCHs, while it has been reported quite comparable rates of reduced ΔΔTSH values in SADs, unipolar, and BD depressed patients (9).

In the present study, we used a series of five neuroendocrine challenges (APO test, CLO test, 8 AM and 11 PM TRH tests, overnight DST) and examined nine hormonal responses in a population of 52 male drug-free hospitalized subjects. Our aim was to identify response patterns in order to provide some indication of altered central nervous system function in patients with psychotic and affective diseases.

## Material and Methods

### Participants

Thirty-nine drug-free male inpatients, without a history of suicidal behavior, and 13 healthy male hospitalized control (HC) subjects participated in this study. Patients were recruited from the inpatient units of the Pole 8/9 of the Centre Hospitalier of Rouffach (France). All subjects underwent a standard clinical interview and a semi-structured diagnostic interview [Schedule for Affective Disorder and Schizophrenia-Lifetime Version (23)]. Patients were independently classified according to the Diagnostic and Statistical Manual of Mental Disorders (DSM-IV) (24) criteria by two psychiatrists, blind to the results of neuroendocrine investigations. The patient group consisted of 13 paranoid SCHs, 13 SADs (bipolar subtype, depressed at the time of the study), and 13 BDs (type II, depressed at the time of the study). Before testing, inpatients were medication-free for at least 2 weeks. The intensity of clinical symptoms was evaluated with the Brief Psychiatric Rating Scale (BPRS, 18-item). The control group consisted of 13 hospitalized normal male volunteers without a personal or family history of major psychiatric illness; none of them met criteria for Axis I diagnostic or had been previously treated with psychotropic medications. This study was approved by the local ethical committee (Rouffach Hospital Review Board), and was conducted in accordance with the Declaration of Helsinki. All subjects gave their informed consent prior to participation.

Routine physical examination and laboratory tests were performed in all subjects. None had a history of endocrinopathy, major medical illness, acute weight change (all were within 15% of ideal body weight), alcohol, or substance abuse. All subjects had basal PRL, TSH, FT_4_, and FT_3_ values within the normal range. No patient had received long-acting neuroleptics, electroconvulsive therapy, lithium salts, fluoxetine, or monoamine oxidase inhibitor antidepressants within 2 years of testing. All subjects were on a caffeine-restricted diet for at least three days before testing and their environment was synchronized, with diurnal activity from 8 AM to 11 PM, and nocturnal rest (sleep).

### Procedures

To reduce bias due to interferences between the tests, the order of the tests was carefully determined. Two TRH-TSH stimulation tests were carried out at 8 AM and 11 PM (day 1), using 200 µg of synthetic TRH IV (Stimu-TSH, Laboratoires Roussel, Paris, France) (25). This procedure has the advantage to take into account the circadian activity of the HPT axis, which is maximal during night. After an overnight fast, subjects were awoken at 7 AM. An indwelling cannula was inserted into an antecubital arm vein and kept open with a slow infusion of 0.9% saline. Baseline blood samples for levels of TSH were collected at -15 and 0 min. The first TRH-TSH stimulation test was carried out at 8 AM, and blood samples were taken after 15, 30, and 60 min. The second TRH-TSH test was performed at 11 PM, on the same day, using the same procedure; subjects were awake during the sampling and fasting from 6 PM. The DST was carried out at midnight with oral ingestion of 1 mg of dexamethasone (Dectancyl, Laboratoires Roussel, Paris, France), followed by blood samples drawn for the assay of serum cortisol at 8 AM, 4 PM, and 11 PM the next day (day 2) (26).

On day 4, an APO test (SC injection of 0.75 mg Apokinon, Laboratoires Aguettant, France) (10) and on day 8, a CLO test (0.375 mg of Catapressan^®^, given orally, Laboratoires Boehringer Ingelheim, France) (27) were carried out at 9 AM, after an overnight fasting, according to the same sampling procedure. Subjects were awoken at 7 AM, and a cannula was inserted into an anterior forearm vein. Blood was drawn at -30, -15, and 0 min before APO or CLO administration and further samples for the assay of GH (following APO and CLO), and PRL, ACTH, cortisol (following APO) were collected at 15, 30, 60, 90, 120, and 150 min. Throughout the tests subjects were in bed and did not smoke.

### Assays

Blood samples were centrifuged at 3,000 rpm and 4°C, and the serum separated and stored at -20°C until assay. All hormone concentrations were determined by immunoassay techniques based on enhanced luminescence (13). The ACTH assay (Nichols Advantage^®^ ACTH, Nichols Institute Diagnostics, San Juan Capistrano, CA) had intra-assay and inter-assay coefficients of variation of 2.7%–7.9% respectively; the sensitivity was 1 ng/l. The GH assay (Nichols Advantage^®^ hGH, same supplier) had intra-assay and inter-assay coefficients of variation of 3.9%–7.5% respectively; the sensitivity was 0.1 µg/l. The TSH assay (Amerlite TSH-60 Assay, Amersham International plc, Amersham, UK) had intra-assay and inter-assay coefficients of variation of 5.1%–7% respectively; the sensitivity was less than 0.04 mU/l. The FT4 assay (Amerlite FT4 Assay, same supplier) had intra-assay and inter-assay coefficients of variation of 5.1%–5.3% respectively; the sensitivity was 0.5 pmol/l. The FT3 assay (Amerlite FT3 Assay, same supplier) had intra-assay and inter-assay coefficients of variation of 6.0%–8.0% respectively; the sensitivity was less than 0.5 pmol/l. The prolactin assay (Amerlite Prolactin Assay, same supplier) had intra-assay and inter-assay coefficients of variation of 5.5%–6%, respectively; the sensitivity was less than 1.3 µg/l.

The cortisol assay (Amerlite Cortisol Assay, same supplier) had intra-assay and inter-assay coefficients of variation of 6.2%–8.9%; the sensitivity was less than 3 nmol/l.

### Statistical Analysis

Hormonal concentrations at 0 min, immediately before SC injection of APO, were used to define baseline values of PRL, ACTH, and cortisol (i.e., PRL*BL*, ACTH*BL*, and cortisol*BL*) (5). ACTH and cortisol responses were determined for each subject by subtracting the baseline level from the peak level after APO (i.e., ΔACTH and Δcortisol). The PRL response to APO was expressed as percentage of change from baseline according to the formula: PRL_S_ = (PRL_S_ *AUC/*PRL*BLAUC*)x100 (10) in which PRL*BLAUC* is the basal PRL area under the curve (calculated as follows: PRL*BL* x 150 min), and PRL_S_ *AUC* is the PRL suppression area (defined as the difference between PRL*BLAUC* and PRL*AUC* after APO). GH values from time points -30, -15, and 0 min were averaged to obtain a single baseline value before APO (GH_APO_ *BL*) and CLO (GH_CLO_ *BL*) stimulation tests. To be included in this research, subjects had to have, before APO and CLO, a GH*BL* value < 2 µg/l. The maximum GH responses to APO and CLO (ΔGH_APO_ and ΔGH_CLO_, respectively) were determined for each subject by subtracting the baseline GH level from the peak GH level. The mean of the two TSH values, at -15 and 0 min, was calculated to give baseline TSH (TSH*BL*) value. The maximum TSH response (ΔTSH) was determined by subtracting TSH*BL* level from the peak TSH level after TRH injection; ΔΔTSH was defined as the difference between 11 PM-ΔTSH and 8 AM-ΔTSH values. To evaluate the cortisol response to DST we used the maximum cortisol level after DST in any blood sample obtained at 8 AM, 4 PM, and 11 PM on day 2 (26).

Analyses were performed using StatView software version 5.0 (SAS Institute Inc, Cary NC, USA). Given the small sample size, non-parametric statistical methods were employed. The comparisons between different patient groups and the control group were performed using the Mann-Whitney two-tailed test (U test)—formal corrections for multiple comparisons were not needed since we made planned comparisons. Within-group differences were tested by the Wilcoxon two-tailed signed rank test (T test) for paired data. Correlations between quantitative variables were estimated using the Spearman rank coefficient (Δ). We used receiver operating characteristic (ROC) curves to determine thresholds of abnormal results (28). Categorical data were analyzed with either Fisher’s exact test (two-tailed) or Yates’ χ^2^-test. Results were considered significant when p ≤ 0.05.

### Results

**Table 1** displays the demographic data and the main results of the DST, TRH, APO, and CLO tests for patients and HCs. Patients and HCs were comparable for age. Basal hormone values were not different across diagnostic groups of subjects. BDs had lower BPRS scores (mean ± SD, 44.6 ± 12.1) than SCHs (54.9 ± 14.9) and SADs (52.7 ± 15.6) (p < 0.05 by U test).

**Table 1:** Demographic characteristics and biological data for normal controls and patients.

	Control subjects(n =13)	Schizophrenic patients(paranoid subtype)(n=13)	Schizoaffective patients(bipolar subtype)(n=13)	Bipolar patients(depressed)(n=13)
Age, years^a^	33.2 ± 9.2	31.1 ± 10.3	32.3 ± 10.8	34.3 ± 10.8
**Apomorphine test**				
PRL*BL* (µg/l)	9.2 ± 5.2	7.0 ± 3.5	8.7 ± 4.4	7.7 ± 3.3
PRL*_S_* (%)	40 ± 16	35 ± 15	24 ± 18*	19 ± 10**
ACTH*BL* (ng/l)	27.5 ± 18.9	25.7 ± 16.1	28.1 ± 15.5	27.2 ± 11.2
ΔACTH (ng/l)	50 ± 74	12.5 ± 26*	51 ± 50	23 ± 38
Cortisol*BL* (nmol/l)	257 ± 75	343 ± 150	332 ± 103	243 ± 81
ΔCortisol (nmol/l)	154 ± 160	26 ± 112*	126 ± 155	107 ± 106
GH*BL* (µg/l)	0.4 ± 0.3	0.7 ± 0.5	1.0 ± 0.8	0.5 ± 0.4
ΔGH (µg/l)	16.6 ± 9.4	15.9 ± 21.0	20.2 ± 17.0	14.3 ± 8.7
**Clonidine test**				
GH*BL* (µg/l)	0.4 ± 0.3	0.6 ± 0.3	0.5 ± 0.4	0.4 ± 0.3
ΔGH (µg/l)	17.4 ± 7.8	15.5 ± 19.4	7.5 ± 10.3**	9.2 ± 8.3*
**TRH tests**				
8 AM-FT4*BL* (pmol/l)	14.9 ± 3.9	15.1 ± 4.2	14.6 ± 4.0	14.3 ± 4.1
8 AM-FT3*BL* (pmol/l)	5.1 ± 0.8	5.2 ± 0.9	5.3 ± 0.8	5.5 ± 0.7
8 AM-TSH*BL* (mU/l)	1.13 ± 0.45	1.30 ± 0.69	1.27 ± 0.50	1.25 ± 0.58
8 AM-ΔTSH (mU/l)	6.6 ± 3.3	6.6 ± 3.8	5.7 ± 2.5	7.4 ± 3.2
11PM-TSH*BL* (mU/l)	1.23 ± 0.72	1.39 ± 0.80	1.18 ± 0.52	0.99 ± 0.55
11 PM-ΔTSH (mU/l)	10.4 ± 4.1	10.4 ± 4.8	7.2 ± 2.4*	8.2 ± 3.4
ΔΔTSH (mU/l)	3.8 ± 1.4	3.8 ± 2.3	1.4 ± 1.3**	0.7 ± 1.4***
**Post-dexamethasone**				
Maximum Cortisol (nmol/l)	26 ± 15	86 ± 124*	64 ± 67*	71 ± 91**

a Values are mean ± SD. PRL indicates, prolactin; ACTH, adrenocorticotropin hormone; GH, growth hormone; TSH, thyrotropin; BL, basal concentration; PRLs, prolactin suppression; Δ, peak concentration minus basal concentration; ΔΔTSH, 11-ΔTSH minus 8 AM-ΔTSH.

Comparisons between control and patient groups were tested by U test (two-tailed): *p ≤ 0.05; **p ≤ 0.01; ***p ≤ 0.001.

### Apomorphine Test

#### PRL Levels

**Figure 1:**
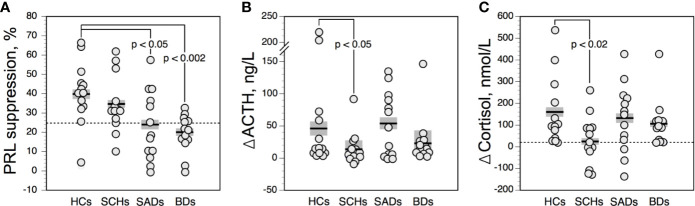
Prolactin suppression **(A)**, and maximum increment in serum adrenocorticotropic hormone (ACTH) **(B)**, and cortisol **(C)** above baseline after 0.75 mg SC of apomorphine in controls and patients. The solid horizontal lines indicate the group mean; the shaded areas represent ± SEM. HCs, healthy control subjects; SCHs, patients with paranoid schizophrenia; SADs, patients with schizoaffective disorder; BDs, patients with bipolar depression.

There was no age effect for PRL*BL* and PRLs values. Compared with HCs, PRL_S_ values were lower in SADs and BDs, while in SCHs the difference was not significant. PRLs values were neither influenced by PRL*BL* levels nor by HPA axis activity (i.e., cortisol at baseline and following DST). As illustrated in **Figure 1A**, 3 SCHs (23%), 7 SADs (54%), 8 BDs (61%), and 1 HC (8%) exhibited a PRLs value below 25%. SADs and BDs showed more frequently blunted PRLs values than HCs (p = 0.03 and p = 0.01, respectively, by Fisher’s exact test). The distribution was not significantly different between SCHs and HCs (p > 0.30 by Fisher’s exact test).

#### ACTH Levels

ACTH values were not related to age. The ACTH response to APO was not correlated with ACTH*BL* levels. Compared with HCs, ΔACTH values (**Figure 1B**) were lower in SCHs, while in SADs and BDs ΔACTH values were not significantly different. Owing to a wide variation of ΔACTH values, no meaningful threshold for a blunted response could be defined.

#### Cortisol Levels

Cortisol*BL* and ΔCortisol values were not influenced by age. ΔCortisol values were lower in SCHs than in HCs. In SADs and BDs, ΔCortisol levels were not significantly altered. Cortisol response to APO was unrelated to the HPA axis activity, as evaluated by cortisol values at baseline and following DST. We found a positive correlation between ΔCortisol and ΔACTH values in the overall population (ρ = 0.75; n = 52; p < 0.00001), in SCHs (ρ = 0.78; n = 13; p = 0.006), in SADs (ρ = 0.68; n = 13; p = 0.01), in BDs (ρ = 0.69; n = 13; p = 0.01), and in HCs (ρ = 0.88; n = 13; p = 0.002). As shown in **Figure 1C**, 7 SCHs (54%), 3 SADs (23%), 3 BDs (23%), and 1 HC (8%) exhibited a ΔCortisol value below 20 nmol/l. While the distribution of blunted cortisol responses was similar in SADs and BDs and did not differ significantly from HCs, blunted cortisol responses were more frequent in SCHs than in HCs (p = 0.03 by Fisher’s exact test).

#### GH Levels

ΔGH_APO_ values did not differ across patients and controls, and were unrelated to age. APO-GH responses were not significantly correlated with GH*BL* levels. Interestingly, ΔGH_APO_ values were positively correlated with ΔACTH and ΔCortisol values in the whole population (ρ = 0.44; n = 52; p = 0.001 and ρ = 0.31; n = 52; p = 0.02, respectively), whereas such a correlation was not found significantly in HCs or in patients. When using a ΔGH_APO_ value of less than 6 µg/l to define a blunted response, 6 SCH (46%), 4 SADs (31%), 2 BDs (15%), and 1 HC (8%) showed blunted responses. Compared to HCs, there was a trend towards increased frequency of blunted GH_APO_ response in SCHs (p = 0.07 by Fisher’s exact test).

### Clonidine Test

**Figure 2:**
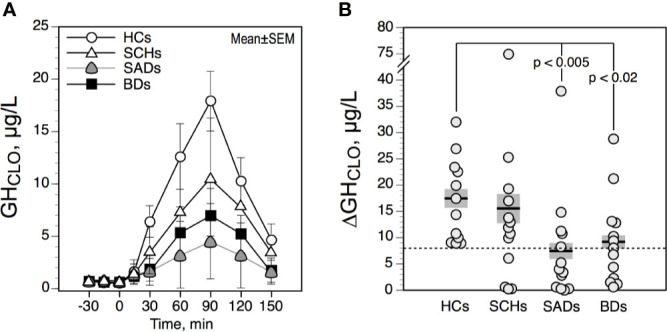
Time course **(A)** and maximum increment **(B)** in serum growth hormone (GH) above baseline after 0.375 mg of clonidine PO in controls and patients. The solid horizontal lines indicate the group mean; the shaded areas represent ± SEM. HCs, healthy control subjects; SCHs, patients with paranoid schizophrenia; SADs, patients with schizoaffective disorder; BDs, patients with bipolar depression.

The GH responses to CLO were not influenced by GH*BL* values. GH*BL* and ΔGH_CLO_ values were not significantly correlated with age in our population. **Figure 2A** shows the time courses of serum GH in the 4 diagnostic groups. ΔGH_CLO_ values were lower in SADs and BDs than in HCs (**Figure 2B**). No such difference was observed between SCHs and HCs. ΔGH_CLO_ and ΔGH_APO_ values were not significantly correlated in the total sample, in patients and in HCs. When using a value of less than 8 µg/l to define a blunted ΔGH_CLO_, 4 SCH (31%), 8 SADs (61%) and 7 BDs (54%) had blunted responses; none were noted in HCs. Blunted GH_CLO_ response was more frequent in SADs and BDs than in HCs (p = 0.01 and p = 0.03 respectively, by Fisher’s exact test), in SCHs the frequency did not reach statistical significance (p = 0.09 by Fisher’s exact test).

### Protirelin (TRH) Tests

**Figure 3:**
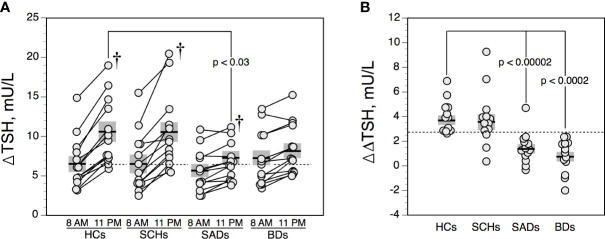
Maximum increment in serum thyrotropin (TSH) level above baseline (ΔTSH) after 200 μg IV of protirelin (TRH) **(A)**, and difference between 11 PM-ΔTSH and 8 A-ΔTSH (ΔΔTSH) **(B)** in controls and patients. The solid horizontal lines indicate the group mean; the shaded areas represent ± SEM. HCs, healthy control subjects; SCHs, patients with paranoid schizophrenia; SADs, patients with schizoaffective disorder; BDs, patients with bipolar depression. ^†^p < 0.005 by T test (comparison between 8 AM-ΔTSH and 11 PM-ΔTSH values).

The effect of age was not significant for FT4, FT3, and TSH values (TSH*BL*, ΔTSH, ΔΔTSH). As illustrated in **Figure 3A**, ΔTSH values were higher in the evening than in the morning in HCs, SCHs and SADs (all p < 0.005 by T test). In BDs, this increment was not significant (p = 0.09 by T test). TRH-TSH responses (i.e., 8 AM-ΔTSH and 11 PM-ΔTSH), when compared with HCs, were not different in SCHs and BDs. In SADs, however, 11PM-ΔTSH values were lower than in HCs. When using an 11PM-ΔTSH value below 6.5 mU/l to define a blunted response, 6 SADs (46%) and 6 BDs (46%) (both p = 0.07 by Fisher’s exact test, when compared with HCs); 3 SCHs (23%) and 1 HC (8%) exhibited a blunted response. As shown in **Figure 3B**, ΔΔTSH values were reduced in SADs and BDs, while SCH showed similar ΔΔTSH values than HCs. Moreover, 12 SADS (92%) and 13 BDs (100%)—while only 2 SCHs (15%) and 1 HC (8%)—exhibited a ΔΔTSH value below 2,5 mU/l. Rates of reduced ΔΔTSH values were comparable in SADs and BDs and were higher than in HCs and SCHs (all p < 0.0003 by Fisher’s exact test).

### Dexamethasone Suppression Test

**Figure 4:**
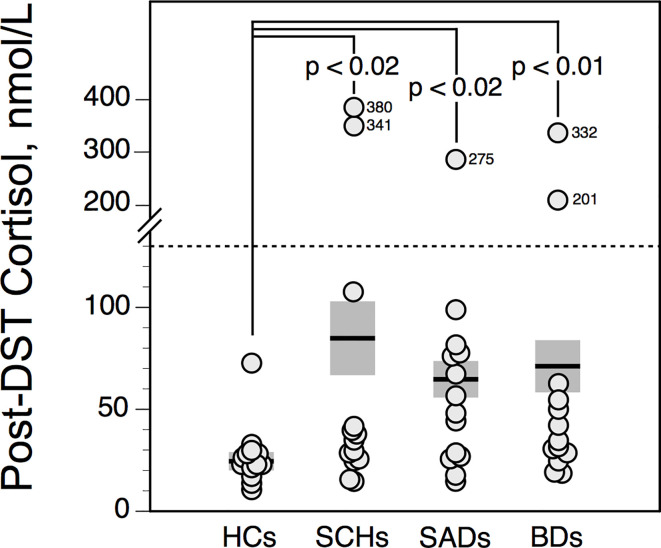
Highest serum cortisol value following dexamethasone suppression test (DST) in controls and patients. The solid horizontal lines indicate the group mean; the shaded areas represent ± SEM. HCs, healthy control subjects; SCHs, patients with paranoid schizophrenia; SADs, patients with schizoaffective disorder; BDs, patients with bipolar depression.

Post-DST cortisol values were not influenced by age. Compared with HCs, post-DST cortisol levels were higher in patients. However the incidence of nonsuppression of cortisol after dexamethasone [i.e., highest post-DST cortisol level > 130 nmol/l (13)] was rather low: DST nonsuppression was observed in 2 SCHs and 2 BDs (both 15%); 1 SAD (8%); and none HC (**Figure 4**).

### Frequency of Abnormal Test Responses Among Patients and Control Subjects

**Figure 5:**
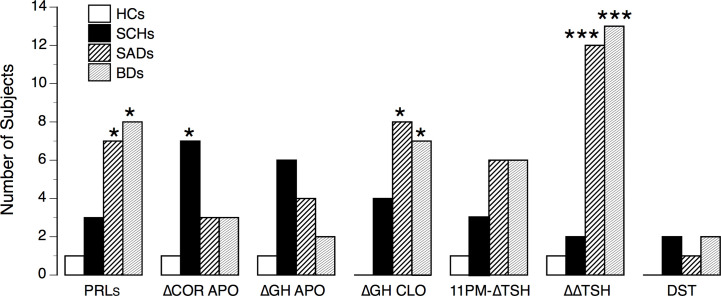
Number of abnormal test responses in controls and patients. HCs, healthy control subjects; SCHs, patients with paranoid schizophrenia; SADs, patients with schizoaffective disorder; BDs, patients with bipolar depression. PRLs, prolactin suppression following apomorphine (APO); ΔCOR APO, maximum increment in serum cortisol level above baseline after APO; ΔGH APO, maximum increment in serum growth hormone (GH) level above baseline after APO; ΔGH CLO, maximum increment in serum growth hormone (GH) level above baseline after clonidine (CLO); 11 PM-ΔTSH, maximum increment in serum thyrotropin level above baseline (ΔTSH) after protirelin (TRH); ΔΔTSH, difference between 11 PM-ΔTSH and 8 A-ΔTSH values. Comparisons between HCs and patients: *p < 0.05; ***p < 0.001 (by Fisher’s exact test).

**Figure 5** summarizes the number of abnormal test responses in patients and HCs. When analyzing the frequency of normal/abnormal test responses (i.e., APO-PRLs, APO-ΔCortisol, CLO-ΔGH, TRH-ΔΔTSH), SADs and BDs displayed a similar pattern of abnormalities, significantly different from SCHs (Yates’ χ^2 =^ 28.43, df =7, p < 0.0002).

## Discussion

Our study clearly demonstrates that multihormonal responses to a series of neuroendocrine test battery (APO test, CLO test, 8 AM and 11 PM TRH tests, and DST) vary according to diagnostic categories. In unmedicated paranoid SCH inpatients, pituitary-adrenal response to APO (i.e., ΔACTH and ΔCortisol) was reduced, while hormone responses to CLO and TRH tests were not significantly altered. The patterns of abnormality of hormonal responses of unmedicated depressed SAD and BD inpatients were very close and were characterized by a reduced APO-induced PRL suppression, a reduced CLO-induced GH stimulation, and a chronobiological alteration of the HPT axis (as reflected by reduced ΔΔTSH values). It should be noted that in the affective groups, BDs showed weaker psychotic symptoms than SADs (as reflected by lower BPRS scores), while their hormonal profile was quite comparable. Hence, this would suggest that the biological correlates of psychotic symptoms depend on the nosographical context. Increased HPA axis activity (as evidenced by higher post-DST cortisol values compared to HCs) was observed in SCHs as well as SADs and BDs—although overt hyperactivity of this axis (i.e., DST non-suppression) was rather infrequent in patients of our sample. In addition, the differences observed in test responses between patients and HCs did not seem to be an artifact of factors known to influence serum hormone levels (such as age, gender, medication) since we investigated a population of middle-aged male drug-free subjects.

### Apomorphine Test

Confirming our previous studies (5, 7, 8), ACTH and cortisol responses to APO are strongly correlated. This suggests that cortisol stimulation by APO, despite localization of DA-D_2_ receptors in the adrenal gland (29), is secondary to that of ACTH. Blunted ACTH/cortisol to APO response has consistently been found in schizophrenia (5–8). This blunting appears independent of HPA axis activity (6) and DST status (7, 8). Moreover, it seems unlikely that decrease APO-induced ACTH/cortisol stimulation is due to decreased reserve of pituitary ACTH (8) or residual antipsychotic effect, given that, in our study, baseline PRL levels are similar between SCHs and HC (antipsychotics *via* a D_2_ blocking effect can increase prolactinemia). From a pathophysiological viewpoint, the mechanisms underlying a reduced ACTH/cortisol response to APO are not completely understood. It is known that APO binds the D_2_-like (D_2_, D_3_, D_4_) receptor and the D_1_-like receptor (D_1_, D_5_) subtypes (30). Since D_2_ and D_1_ receptors are involved in the regulation of CRH (31, 32)—and therefore ACTH release—one may hypothesize that the blunted ACTH/cortisol response to APO reflects reduced hypothalamic DA receptor sensitivity. Interestingly, D_2_ receptors are also expressed in the pituitary corticotroph cells but their role is thought to be inhibitory on ACTH secretion (33). Therefore, the blunted ACTH/cortisol response to APO in paranoid SCHs is compatible with a hyposensitivity (or persistent down-regulation) of the DA-D_2_ and/or D_1_ receptors connected with the regulation of HPA axis possibly secondary to increased presynaptic DA activity at the hypothalamic level. Given the DA abnormality in SCH is thought to primarily involve synthesis and release activity (34), our results are in line with the hypothesis of an increased DA activity in the mesolimbic-hypothalamic pathway in paranoid SCHs. However, APO has also affinity for serotonin receptors (5-HT_1A_, 5-HT_2A_, 5-HT_2B_, and 5-HT_2C_), and α-adrenergic receptors (α_1B_, α_1D_, α_2A_, α_2B_, and α_2C_) (30, 35). Most of these receptors have been involved to different degrees in the regulation of CRH activity [for a review, see (3)]. Consequently, the blunted APO-induced ACTH/cortisol stimulation might also reflect in part 5-HT and α-adrenergic receptor dysfunction, although this hypothesis needs further investigation in schizophrenic patients.

The GH and ACTH/cortisol responses to APO are correlated in the whole sample, but not in the diagnostic groups of subjects. Despite there is a trend towards blunting in SCHs, ΔGH_APO_ values are not significantly different across patients and HCs. This latter result is in agreement with previous published reports (5–7, 36, 37) but not all (38, 39). As previously discussed (8), the effect of APO on GH involves different pathways from those mediating ACTH/cortisol response, since this response requires the participation of GH-releasing-hormone (GHRH) neurons and acetylcholine, and other neurotransmitters/hormones such as NA, 5-HT, GABA, ghrelin, and cholecystokinin are probably involved in the GH response to APO. These confounding factors consequently may limit the value of the GH response to APO in the investigation of DA function in psychiatry.

In agreement with several studies (5, 9, 12, 40) APO induced-PRL suppression is altered in our population of BDs and SADs. The lack of significant difference in the PRL response to APO between SCHs and HCs is also consistent with prior reports (5, 7, 9) but not all (41). The release of PRL is inhibited by the tuberoinfundibular (TI) DA neurons *via* D_2_ receptors (4). Our findings suggest hyposensitivity of the D_2_ receptors of the lactotrophs in BDs and SADs, possibly secondary to the activation of the TIDA neurons. However, it is also possible that PRLs blunting might be due to functional alteration of lactotrophs cells. This hypothesis is not confirmed by a previous study (40), in which 8 AM and 11 PM PRL responses to TRH stimulation tests were comparable between unipolar (UP) and bipolar depressed patients, while BDs, unlike UPs, exhibited blunted APO-induced PRLs values (12).

### Clonidine Test

CLO induces a robust GH response *via* activation of postsynaptic α_2_-adrenoceptors, which increase the secretion of GHRH and inhibit the secretion of somatostatin (42). The blunted GH response to CLO is well documented in depression (16, 26, 43) and in SAD (9, 16). Such a response may be due to decreased postsynaptic α_2_-receptor responsiveness linked to an erratic release of presynaptic NA (43). Thus, the comparable ΔGH values found in depressed BDs and SADs suggest a possible biological link between these two diseases (i.e., NA dysregulation). In agreement with a previous study of our group (16), ΔGH_CLO_ values in paranoid SCHs are not altered (suggesting normal sensitivity of hypothalamic α_2_-adrenoreceptors in these patients), although in disorganized SCHs it has been found greater CLO-induced GH responses (37) (suggesting hypersensitivity of α_2_-adrenoreceptors in these patients). However, this latter finding has not been replicated (16).

### Protirelin (TRH) Tests

Results obtained from the morning TRH-TSH challenge agree with those of previous published reports [for review, see (21)]. Morning TRH-TSH responses are not significantly different across the patient and control groups. In the evening, TRH-TSH responses at 11 PM are higher than at 8 AM (albeit not significant in BDs). Consistent with a previous study, ΔΔTSH values are reduced in depressed SADs and BDs, while they are unaltered in SCHs (9). We have already discussed that the ΔΔTSH test is a chronobiological refinement of the TRH test (25, 44). Pathophysiological components involved in an abnormal ΔΔTSH test may be synthesized as follows (21):

1. A chronobiological component involving the determinants of circadian TSH secretion [i.e., a weaker output of the hypothalamic suprachiasmatic nuclei (45)], since reduced ΔΔTSH values are associated with decreased 24-h TSH mesor and amplitude levels in depression (25).
2. A chronesthesic component involving TRH receptor sensitivity, since altered sensitivity of TRH receptors is more evidenced at 11 PM than at 8 AM (25). TRH receptor hyposensitivity may be adaptive to prolonged hypersecretion of endogenous TRH (46).
3. A self-regulating component, since the ΔΔTSH test takes into account the negative feedback of thyroid hormones on TSH secretion. Indeed, the TRH test performed at 8 AM stimulates thyroid hormone secretion, increasing, therefore, the negative feedback of thyroid hormones on TSH secretion in the evening (44).
4. A dynamic component, since 11 PM-ΔTSH blunting could also be related to a reduced TSH resynthesis in the thyrotrophs during the hours following the 8 AM TRH test (given that TRH stimulates preformed TSH). Decreased TSH synthesis could involve a hyposensitivity of the pituitary TRH receptors and/or an increased negative feedback of thyroid hormones, or a decreased central TRH activity [especially in recent suicide attempters in whom FT_4_ levels are also reduced (44)]. In our population, no patient had a history of suicidal behavior; therefore reduced ΔΔTSH values in SADs and BDs are unlikely to be due to a decrease in the central activity of TRH.

### Dexamethasone Suppression Test

In our sample, SCHs, SADs and BDs exhibit significant higher post-DST cortisol values than HCs, indicating a weaker suppressing effect of dexamethasone. This finding, which could reflect decreased type II GR function, converges with the growing literature on HPA axis dysregulation in psychotic and affective diseases (18, 19, 47). However in our study, DST nonsuppression occurs only in a low proportion of patients. This non-expected low incidence—especially in depressed SADs and BDs—is nonetheless in accordance with some previous but not all reports [for review, see (3)]. We could presume that the sensitivity in detecting HPA axis overactivity would be better using the combined dexamethasone/corticotropin-releasing hormone (DEX/CRH) test (48–50), although all studies do not agree (51, 52). It has been hypothesized that the hyperactivity of the HPA axis is primarily a reflection of abnormal limbic-hypothalamic activation, with increased secretion of hypothalamic CRH and consequent excessive adrenal cortisol secretion (17). Given the high rate of reduced ΔΔTSH values in BDs and SADs—possibly reflecting endogenous TRH hypersecretion—one may hypothesize that increased TRH secretion (both from hypophysiotropic and non-hypophysiotropic neurons) could decrease glucocorticoid secretion by impairing the last steps of 11ß-hydroxylation without affecting the earlier steps (53). In such case, the GR function would be only partially attenuated, despite CRH overdrive, explaining therefore why SAD and BD patients with reduced ΔΔTSH values are often DST suppressors.

### Limitations

Some shortcomings in this present study require discussion. First, our results concern only a specific group of drug-free male inpatients; they do not appear at present transposable to outpatients, and consequently they cannot be generalizable to affective and psychotic patients. Second, given the exploratory nature of our research, and the drastic inclusion criteria, we studied a rather small sample of psychiatric inpatients. This may have reduced the statistical power of our analyses (performed with nonparametric methods). Thus, our findings must be considered preliminary until replicated in a larger patient population. Third, among the confounding factors in assessing neurotransmitter function, insufficient washout period could be a major bias. However, our exclusion criteria and the length of the wash-out period (minimum 2 weeks for the APO test and 3 weeks for the CLO test) seem sufficient to avoid biases induced by drugs on the systems studied (5, 54). Finally, we did not measure serum dexamethasone. However, it has been argued that the concentration of dexamethasone bound to the receptors in the pituitary is the relevant physiologic parameter rather than the dexamethasone concentration in plasma (55, 56).

In conclusion, the multivariate neuroendocrine approach used in this study was able to identify patterns of hormonal response abnormalities in drug-free hospitalized patients with psychotic and affective symptoms. From a pathophysiological viewpoint, our results suggest that depressed bipolar and schizoaffective patients share common biological dysregulations, clearly distinct from that of paranoid schizophrenic patients. Future studies are needed to determine whether these findings could be relevant in managing psychiatric treatments.

## Data Availability Statement

The datasets generated for this study are available on request to the corresponding author.

## Ethics Statement

The studies involving human participants were reviewed and approved by Centre Hospitalier Rouffach. The patients/participants provided their written informed consent to participate in this study.

## Author Contributions

FD designed the study, wrote the protocol, and wrote the first draft of the manuscript. M-CM undertook the statistical analysis and interpreted the results. AE made clinical assessments. FG made clinical assessments. VD made clinical assessments. LJ managed the literature searches. All authors contributed to the article and approved the submitted version.

## Funding

Funding of this study was provided by inner hospital sources (Centre Hospitalier, Rouffach). No outside parties had any role in study design; in the collection, analysis, and interpretation of data; in the writing of the report and in the decision to submit the paper for publication.

## Conflict of Interest

The authors declare that the research was conducted in the absence of any commercial or financial relationships that could be construed as a potential conflict of interest.

## Abbreviations

ACTH, adrenocortcotropic hormone; APO, apomorphine; BD, bipolar disorder; CLO, clonidine; DA, dopamine; DST, dexamethasone suppression test; GH, growth hormone; HPA, hypothalamic-pituitary adrenal (axis); HPT, hypothalamic-pituitary thyroid (axis); 5-HT, serotonin (5-hydroxytryptamine); NA, noradrenaline; PRL, prolactin; SAD, schizoaffective Disorder; SCH, schizophrenia; TRH, thyrotropin-releasing hormone; TSH, thyroid-stimulating hormone (thyrotropin).

## References

[1] Ashok AH Marques TR Jauhar S Nour MM Goodwin GM Young AH The dopamine hypothesis of bipolar affective disorder: the state of the art and implications for treatment. Mol Psychiatr (2017) 22:666–79. 10.1038/mp.2017.16 PMC5401767 28289283

[2] Yang AC Tsai SJ New targets for schizophrenia treatment beyond the dopamine hypothesis. Int J Mol Sci (2017) 18:E1689. 10.3390/ijms18081689 28771182 PMC5578079

[3] Duval F Endocrinologie et Psychiatrie [Article in French]. EMC Psychiatr (2016) 13(4):1–27. 10.1016/S0246-1072(16)75332-6

[4] Lal S Apomorphine in the evaluation of dopaminergic function in man. Prog Neuro-Psychopharmacol Biol Psychiatry (1988) 12:117–64. 10.1016/0278-5846(88)90033-4 3290992

[5] Mokrani MC Duval F Crocq MA Bailey PE Macher JP Multihormonal responses to apomorphine in mental illness. Psychoneuroendocrinology (1995) 20:365–75. 10.1016/0306-4530(94)00065-4 8532820

[6] Meltzer HY Lee MA Jayathilake K The blunted plasma cortisol response to apomorphine and its relationship to treatment response in patients with schizophrenia. Neuropsychopharmacology (2001) 24:278–90. 10.1016/S0893-133X(00)00201-3 11166518

[7] Duval F Mokrani MC Crocq MA Bailey P Diep TS Correa H Dopaminergic function and the cortisol response to dexamethasone in psychotic depression. Prog Neuro-Psychopharmacol Biol Psychiat (2000) 24:207–25. 10.1016/S0278-5846(99)00098-6 10800744

[8] Duval F Mokrani MC Monréal J Bailey P Valdebenito M Crocq MA Dopamine and serotonin function in untreated schizophrenia: clinical correlates of the apomorphine and d-fenfluramine tests. Psychoneuroendocrinology (2003) 28:627–42. 10.1016/S0306-4530(02)00047-1 12727131

[9] Duval F Mokrani MC Crocq MA Jautz M Bailey PE Diep TS Multihormonal reponses to a series of neuroendocrine challenges in psychiatry: a multivariate approach. In: Macher JP Crocq MA Nedelec JF, editors. New Prospects in Psychiatry: The Bio-clinical Interface. Paris, F: John Libbey Eurotext (1995). p. 77–90.

[10] Meltzer HY Kolakowska T Fang VS Fogg L Robertson A Lewine R Growth hormone and prolactin response to apomorphine in schizophrenia and the major affective disorders. Arch Gen Psychiatry (1984) 41:512–9. 10.1001/archpsyc.1984.01790160098013 6721674

[11] Muller-Spahn F Modell S Ackenheil M Brachner A Kurtz G Elevated response of growth hormone to graded doses of apomorphine in schizophrenic patients. J Psychiatr Res (1998) 32:265–71. 10.1016/S0022-3956(98)00005-3 9789204

[12] Monreal JA Duval F Mokrani MC Fattah S Palao D Differences in multihormonal responses to the dopamine agonist apomorphine between unipolar and bipolar depressed patients. J Psychiatr Res (2019) 112:18–22. 10.1016/j.jpsychires.2019.02.009 30836201

[13] Duval F Mokrani MC Monréal J Fattah S Champeval C Schulz P Cortisol hypersecretion in unipolar major depression with melancholic and psychotic features : dopaminergic, noradrenergic and thyroid correlates. Psychoneuroendocrinology (2006) 31:876–88. 10.1016/j.psyneuen.2006.04.003 16769179

[14] Schatzberg AF Rothschild AJ Langlais PJ Bird ED Cole JO A corticosteroid/dopamine hypothesis for psychotic depression and related states. J Psychiatr Res (1985) 19:57–64. 10.1016/0022-3956(85)90068-8 2859366

[15] Siever LJ Uhde TW New studies and perspectives on the noradrenergic receptor system in depression: effect of the α2-adrenergic agonist clonidine. Biol Psychiatry (1984) 19:131–56. 6324896

[16] Mokrani MC Duval F Diep TS Bailey PE Macher JP Multihormonal response to clonidine in patients with affective and psychotic symptoms. Psychoneuroendocrinology (2000) 25:741–52. 10.1016/S0306-4530(00)00024-X 10938452

[17] Gillespie CF Nemeroff CB Hypercortisolemia and depression. Psychosom Med (2005) 67 Suppl 1:S26–28. 10.1097/01.psy.0000163456.22154.d2 15953796

[18] Naughton M Dinan TG Scott LV Corticotropin-releasing hormone and the hypothalamic-pituitary-adrenal axis in psychiatric disease. Handb Clin Neurol (2014) 124:69–91. 10.1016/B978-0-444-59602-4.00005-8 25248580

[19] Cherian K Schatzberg AF Keller J HPA axis in psychotic major depression and schizophrenia spectrum disorders: cortisol, clinical symptomatology, and cognition. Schizophr Res (2019) 213:72–9. 10.1016/j.schres.2019.07.003 31307859

[20] APA Task force on laboratory tests The dexamethasone suppression test: an overview of its current status in psychiatry. Am J Psychiatry (1987) 14:1253–62. 10.1176/ajp.144.10.1253 3310667

[21] Duval F Mokrani MC Thyroid Axis Activity in Depression. Ann Thyroid Res (2018) 4(3):166–71.

[22] Jackson IM The thyroid axis and depression. Thyroid (1998) 8:951–6. 10.1089/thy.1998.8.951 9827665

[23] Endicott J Spitzer RL [Schedule for Affective Disorders and Schizophrenia (SADS)] [Article in French]. Acta Psychiatr Belg (1987) 87(4):361–516. 3434318

[24] American Psychiatric Association Diagnostic and Statistical Manual of Mental Disorders. 4th ed. Washington, DC: American Psychiatric Press (1994). p. 886.

[25] Duval F Macher JP Mokrani MC Difference between evening and morning thyrotropin responses to protirelin in major depressive episode. Arch Gen Psychiatry (1990) 47:443–8. 10.1001/archpsyc.1990.01810170043007 2109971

[26] Carroll BJ Feinberg M Greden JF Tarika J Albala AA Haskett RF A specific laboratory test for the diagnosis of melancholia. Standardization, validation, and clinical utility. Arch Gen Psychiatry (1981) 8:15–22. 10.1001/archpsyc.1981.01780260017001 7458567

[27] Valdivieso S Duval F Mokrani MC Schaltenbrandt N Oliveira Castro J Crocq MA Growth hormone response to clonidine and the cortisol response to dexamethasone in depressive patients. Psychiatry Res (1996) 60:23–32. 10.1016/0165-1781(95)02606-1 8852865

[28] Metz CE Basic principles of ROC analysis. Semin Nucl Med (1978) 8:283–98. 10.1016/S0001-2998(78)80014-2 112681

[29] Pivonello R Ferone D de Herder WW de Krijger RR Waaijers M Mooij DM Dopamine receptor expression and function in human normal adrenal gland and adrenal tumors. J Clin Endocrinol Metab (2004) 89:4493–502. 10.1210/jc.2003-031746 15356054

[30] Millan MJ Maiofiss L Cussac D Audinot V Boutin JA Newman-Tancredi A Differential actions of antiparkinson agents at multiple classes of monoaminergic receptor. I. A multivariate analysis of the binding pro les of 14 drugs at 21 native and cloned human receptor subtypes. J Pharmacol Exp Therapeut (2002) 303:791–804. 10.1124/jpet.102.039867 12388666

[31] Borowski B Kuhn C D1 and D2 dopamine receptors stimulate hypothalamo-pituitary-adrenal activity in rats. Neuropharmacology (1992) 31:671–8. 10.1016/0028-3908(92)90145-F 1328919

[32] Eaton MJ Cheung S Moore KE Lookingland KJ Dopamine receptor-mediated regulation of corticotropin-releasing hormone neurons in the hypothalamic paraventricular nucleus. Brain Res (1996) 738:60–6. 10.1016/0006-8993(96)00765-2 8949928

[33] Pivonello R Waaijers M Kros JM Pivonello C de Angelis C Cozzolino A Dopamine D2 receptor expression in the corticotroph cells of the human normal pituitary gland. Endocrine (2017) 57:314–25. 10.1007/s12020-016-1107-2 27738887

[34] Howes OD Kambeitz J Kim E Stahl D Slifstein M Abi-Dargham A The nature of dopamine dysfunction in schizophrenia and what this means for treatment. Arch Gen Psychiatry (2012) 69:776–86. 10.1001/archgenpsychiatry.2012.169 PMC3730746 22474070

[35] Kvernmo T Hartter S Burger E A review of the receptor-binding and pharmacokinetic properties of dopamine agonists. Clin Ther (2006) 28:1065–78. 10.1016/j.clinthera.2006.08.004 16982285

[36] Malas KL van Kammen DP de Fraites EA Brown GM Gold PW Reduced growth hormone response to apomorphine in schizophrenic patients with poor premorbid social functioning. J Neural Transm (1987) 69:319–24. 10.1007/BF01244352 3625198

[37] Brambilla F Marini S Saito A Fassone G Picardi A Nerozzi D Noradrenergic and dopaminergic interrelation in schizophrenia. Psychiatry Res (1994) 53(3):231–42. 10.1016/0165-1781(94)90052-3 7870845

[38] Cleghorn JM Brown GM Brown PJ Kaplan RD Mitton J Longitudinal instability of hormone responses in schizophrenia. Prog Neuropsychopharmacol Biol Psychiatry (1983) 7:545–9. 10.1016/0278-5846(83)90023-4 6686692

[39] Zemlan FP Hirschowitz J Garver DL Relation of clinical symptoms to apomorphine-stimulated growth hormone release in mood-incongruent psychotic patients. Arch Gen Psychiatry (1986) 43:1162–7. 10.1001/archpsyc.1986.01800120048010 3778112

[40] Monreal J Duval F Mokrani MC Pinault G Macher JP Exploration de la fonction dopaminergique dans les depressions bipolares et unipolares. Ann Med Psychol (2005) 163:399–404. 10.1016/j.amp.2005.04.011

[41] Tamminga CA Smith RC Pandey G Frohman LA Davis JM A neuroendocrine study of supersensitivity in tardive dyskinesia. Arch Gen Psychiatry (1977) 34:1199–203. 10.1001/archpsyc.1977.01770220081009 911219

[42] Al-Damluji S Adrenergic control of the secretion of anterior pituitary hormones. Baillieres Clin Endocrinol Metab (1993) 7:355–92. 10.1016/S0950-351X(05)80180-6 8387773

[43] Siever LJ Davis KL Overview: toward a dysregulation hypothesis of depression. Am J Psychiatry (1985) 142:1017–31. 10.1176/ajp.142.9.1017 2862799

[44] Duval F Mokrani MC Erb A Gonzalez Lopera FG Calleja C Paris V Relationship between chronobiological thyrotropin and prolactin responses to protirelin (TRH) and suicidal behavior in depressed patients. Psychoneuroendocrinology (2017) 85:100–9. 10.1016/j.psyneuen.2017.07.488 28843902

[45] Duval F Mokrani MC Erb A Gonzalez F Danila V Raverot V Relationship between melatonergic and thyroid systems in depression. Endocrinol Diabetes Metab J (2019) 3:1–4. 10.1016/j.euroneuro.2018.11.554

[46] Loosen PT Prange AJ Jr Serum thyrotropin response to thyrotropin-releasing hormone in psychiatric patients: a review. Am J Psychiatry (1982) 139:405–16. 10.1176/ajp.139.4.405 6802002

[47] Jacobson L Hypothalamic-pituitary-adrenocortical axis: neuropsychiatric aspects. Compr Physiol (2014) 4:715–38. 10.1002/cphy.c130036 24715565

[48] Heuser I Yassouridis A Holsboer F The combined dexamethasone/ CRH test: a refined laboratory test for psychiatric disorders. J Psychiatr Res (1994) 28:341–56. 10.1016/0022-3956(94)90017-5 7877114

[49] Watson S Gallagher P Smith MS Ferrier IN Young AH The dex/CRH test is it better than the DST? Psychoneuroendocrinology (2006) 31:889–94. 10.1016/j.psyneuen.2006.03.001 16701957

[50] Mokhtari M Arfken C Boutros N The DEX/CRH test for major depression: a potentially useful diagnostic test. Psychiatry Res (2013) 208:131–9. 10.1016/j.psychres.2012.09.032 23291044

[51] Schüle C Baghai TC Eser D Häfner S Born C Herrmann S The combined dexamethasone/CRH Test (DEX/CRH test) and prediction of acute treatment response in major depression. PloS One (2009) 4(1):e4324. 10.1371/journal.pone.0004324 19177168 PMC2629564

[52] Kinoshita S Kanazawa T Kikuyama H Yoneda H Clinical application of DEX/CRH test and multi-channel NIRS in patients with depression. Behav Brain Funct (2016) 12(1):25. 10.1186/s12993-016-0108-x 27582123 PMC5007847

[53] Neri G Malendowicz LK Andreis P Nussdorfer GG Thyrotropin-releasing hormone inhibits glucocorticoid secretion of rat adrenal cortex: in vivo and in vitro studies. Endocrinology (1993) 133:511–4. 10.1210/endo.133.2.8393765 8393765

[54] Schittecatte M Charles G Machowski R Wilmotte J Tricyclic wash-out and growth hormone response to clonidine. Brit J Psychiatry (1989) 154:858–63. 10.1192/bjp.154.6.858 2597894

[55] Wiedemann K Holsboer F The effect of dexamethasone dosage upon plasma cortisol and dexamethasone during the DST. J Affect Disord (1990) 19:133–7. 10.1016/0165-0327(90)90018-4 2142700

[56] Young EA Kotun J Haskett RF Grunhaus L Greden JF Watson SJ Dissociation between pituitary and adrenal suppression to dexamethasone in depression. Arch Gen Psychiatry (1993) 50:395–403. 10.1001/archpsyc.1993.01820170073010 8489328

